# Stretchable Cellulosic Cholesteric Liquid Crystal Filaments with Color Response

**DOI:** 10.1021/acsapm.4c02719

**Published:** 2025-03-26

**Authors:** Hongning Ren, Ifeoluwa Omolola Sodipo, Ahu Gümrah Dumanli

**Affiliations:** † Department of Materials, The University of Manchester, Oxford Rd., Manchester M13 9PL, United Kingdom; ‡ Henry Royce Institute, The University of Manchester, Oxford Rd., Manchester M13 9PL, United Kingdom

**Keywords:** Hydroxypropyl cellulose, stretchable color response, cholesteric liquid crystal, mechanochromic sensing, encapsulation

## Abstract

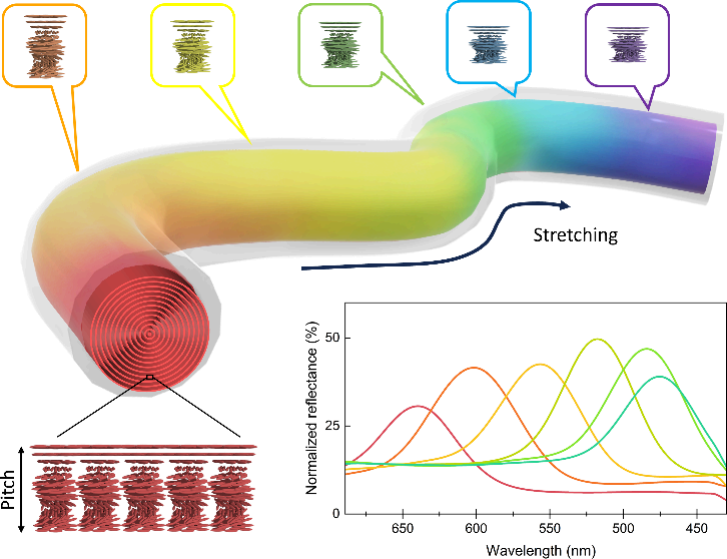

Utilizing soft optical materials in mechanochromic sensing is an intriguing concept with several application areas in soft robotics and functional wearables. Herein, we developed stretchable cholesteric cellulosic liquid crystal filaments by simply encapsulating the aqueous hydroxypropyl cellulose (HPC) mesophases in a sealed thermoplastic elastomeric tubing. The tubular confinement of the HPC induced a well-defined cholesteric structure in which the quasi-layers are concentrically distributed along the fiber axis. The flow properties of the aqueous HPC showcased the ability to mimic the stretching and relaxing behavior of the elastomeric tubing, resulting in a spontaneous color change in response to mechanical deformation. This soft optical system can act as a stretchable colorimetric sensor for both tensile and compression forces enabled by the uniform alignment and dynamic deformation of the HPC mesophase.

## Introduction

Cholesteric liquid crystals (CLCs) have drawn significant attention for mechanochromic sensing due to their helicoidal structure causing chiroptical activity. This helicoidal arrangement is characterized by the cholesteric periodicity, defined by the pitch—i.e., the full 360 deg of cholesteric twist.^1−7^ The relationship between the cholesteric pitch and reflection wavelength is described by de Vries’ equation,
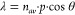
1where n is the average refractive index, θ is the angle of reflection with respect to the cholesteric helix axis, and *p* is the cholesteric pitch.^8,9^ The pitch can be modulated by controlling the environmental conditions, including temperature and humidity, applied external fields such as electric field and mechanical forces, and in lyotropic systems via changing the CLC concentration. Such changes in the pitch cause alteration of the reflected color from the infrared (IR) to the ultraviolet (UV) range.^10−14^

Hydroxypropyl cellulose (HPC) is one of the sought-after CLC systems due to its biocompatibility, cost-efficiency and abundance. HPC as a biopolymer has been widely used in the food and pharmaceutical industries due to its readily film-forming properties and ease of modulating viscosity.^15−19^ HPC can spontaneously form cholesteric mesophases in highly concentrated (over 52 wt %) aqueous solutions. When the solution concentration is between 60 and 70 wt %, it reflects light in the visible range, exhibiting bright colors.^19−21^ The pitch and the reflectance wavelength of HPC are also dependent on the molecular weight, degree of substitution and ionic interactions within the solution.^19,22−25^

In liquid cholesteric form, HPC’s color was shown to have a dynamic response with mechanical stimuli as a compression sensor^26^ or controlled evaporation as an edible timer.^27^ Their further properties, such as, temperature and electrical field response as well as rheological properties, were investigated recently.^28−33^ While these studies demonstrated the significant potential of HPC as a dynamic colorimetric sensing platform, the limited sensitivity of film sheets with respect to weak mechanical and electrical forces restricts their application in electronic display technologies. Furthermore, the trapped gel conformation resulting from the roll-to-roll production of HPC is impractical for functional garment development. Therefore, there is a gap in the processing of the cholesteric HPC mesophases while keeping their dynamic color response for functional garments and soft robotics.

In this work, we produced stretchable filaments of HPC by encapsulating the cholesteric solutions in thermoplastic elastomer tubing and explored the relationship between applied tension and color. By simply extruding the HPC solution into flexible tubes, we fabricated dynamic and stretchable filaments that can act as mechanochromic sensors to measure the distance corresponding to a given strain. Applied tensile forces on the HPC-elastomer tube composite filaments caused a blueshift due to the radial compression of the pitch with applied tensile stress. Furthermore, applying compression onto the filaments also demonstrated a blue shift in color appearance, similar to the laminated HPC constructs shown in the literature,^26^ demonstrating dual color shift functionality (Figure S1 and Video 1).

The prior work on stretchable CLC elastomers where the CLCs were cross-linked with an elastomer matrix provides crucial groundwork for understanding the effect of mechanical strain on the cholesteric liquid crystalline periodicity and pitch.^3,5,34,35^ Our system offers a much simpler, alternative fabrication method as in our work, HPC remains in a fluid state, conserving its dynamic flow properties and allowing a direct analysis of its confinement within the tube. Our microstructural analysis also reveals a cross-sectional morphology of the dried cholesteric structure within the elastomer tubing to conform into a radial concentric layered structure with periodic hierarchical wrinkling caused by Helfrich-Hurault instability due to the relaxation of the kinetically arrested cholesteric phase following shear deformation.^24,36^ In this sense, our work showcases a proof-of-concept prototype using HPC in a broad spectrum of applications, including colorimetric strain sensors for smart fabrics, functional wearables and materials for arts and robotics, opening new horizons for HPC as a functional material.

## Experimental Section

### Material Preparation

Highly concentrated HPC solutions (64.5 wt %) were prepared by mixing HPC powder (SSL grade, MW 40k, Nisso Chemicals, JP) with Milli-Q water (8.2 MΩ cm, Suez Fusion 320). The homogeneous solution was attained by mixing and centrifuging (6,000 rpm/2880 rcf, 5 min), repeated 3–4 times with hourly intervals with the OHAUS FC5718R Frontier refrigerated centrifuge.

### HPC Tubing System Preparation

Before extrusion, the HPC solutions were transferred into printing cartridges and centrifuged for at least 10 min at 6,000 rpm (2880 rcf) to remove air bubbles. This HPC solution was then injected into clear Tygon E3603 tubing (internal diameter 1.6 mm, external diameter 3.2 mm, wall thickness 0.8 mm, Saint-Gobain) through an 18-gauge nozzle using an extrusion pressure of 6 bar supplied by Nordson EFD LLC. 7012334 Performus syringe fluid dispenser, attached to a 3d printer. Subsequently, both ends of the tube were blocked by fitting matched paper rods and sealing epoxy glues (MitreBond Aerosol Kit) to prevent evaporation.

### Visual observation

To demonstrate the color response by stretching, digital images were recorded by a Panasonic LUMIX DMC-LX15 digital camera. The visual appearance of the encapsulated filaments was recorded by producing a 20 cm long tube and taking images from the central 8 cm-10 cm mark.

### Refractive Index Measurement of the HPC Solutions

The average of the ordinary and extraordinary refractive indices of HPC has been carefully measured using a KRÜSS Optronic Analogue AR3 Abbe Refractometer. For the detailed measurement of the refractive index, please refer to Section S1 and Figure S1.

### Spectroscopic Measurements

The optical imaging and reflection measurements were performed by a custom-modified polarized optical microscope (Olympus BX53MTRF) coupled to an Ocean Insight Flame spectrometer connected by an Ocean Insight 50 μm UV–visible optical Fiber. Before the measurements, a 75% Spectralon Diffuse Reflectance Standard was used for calibration. The reflection spectra were smoothed using the LOWESS method and normalized by taking the 50% strain sample at 50% reflectance maxima.

The microscopic measurements were taken from a 40 cm long tube which was fixed to the microscopy stage. To exclude the influence of both sides, only the central 30 cm was used for observation.

The color change as a function of stretching deformation was recorded by fixing the HPC filaments using a G-type clamp (51 mm capacity) on one side and stretching the filament along a stainless-steel ruler. The filament was extended up to 185% of its starting length manually. After this point, the color response was diminished due to a shift to UV color response.

### Microstructure Analysis

The microstructure of the fiber cross-section was imaged using a Zeiss Sigma FEG SEM. To prepare the SEM samples, HPC solution was dried at 60 °C together with the elastomer tubing until all the water was removed. Before the imaging, the HPC fiber construct was taken out of the tubing and sliced at an oblique angle using a razor. The cross-section samples were then placed on the 45/90-degree SEM stubs vertically using carbon tape with the sides wrapped with sticky copper tape. To avoid charging under the SEM, the samples were deposited with 8 nm of Au/Pt coating using a Quorum sputter-coater Q150R Plus (an Au/Pt target 80:20 was used at 40 mA current).

### Mechanical Testing

The mechanical behavior of the fibers was determined by tensile testing on an INSTRON 3345 series universal testing system. The Tygon tubes were cut to a length of 25 cm. Empty and HPC-filled tubes were tested at a gauge length of 75 mm using yarn/cord pneumatic grips. The test was conducted with a 5 kN load cell and a constant rate of extension of 30 mm/min. Each specimen was tested three times and the average elastic moduli were calculated using Python (Table S1).

## Results and Discussion

**Figure 1:**
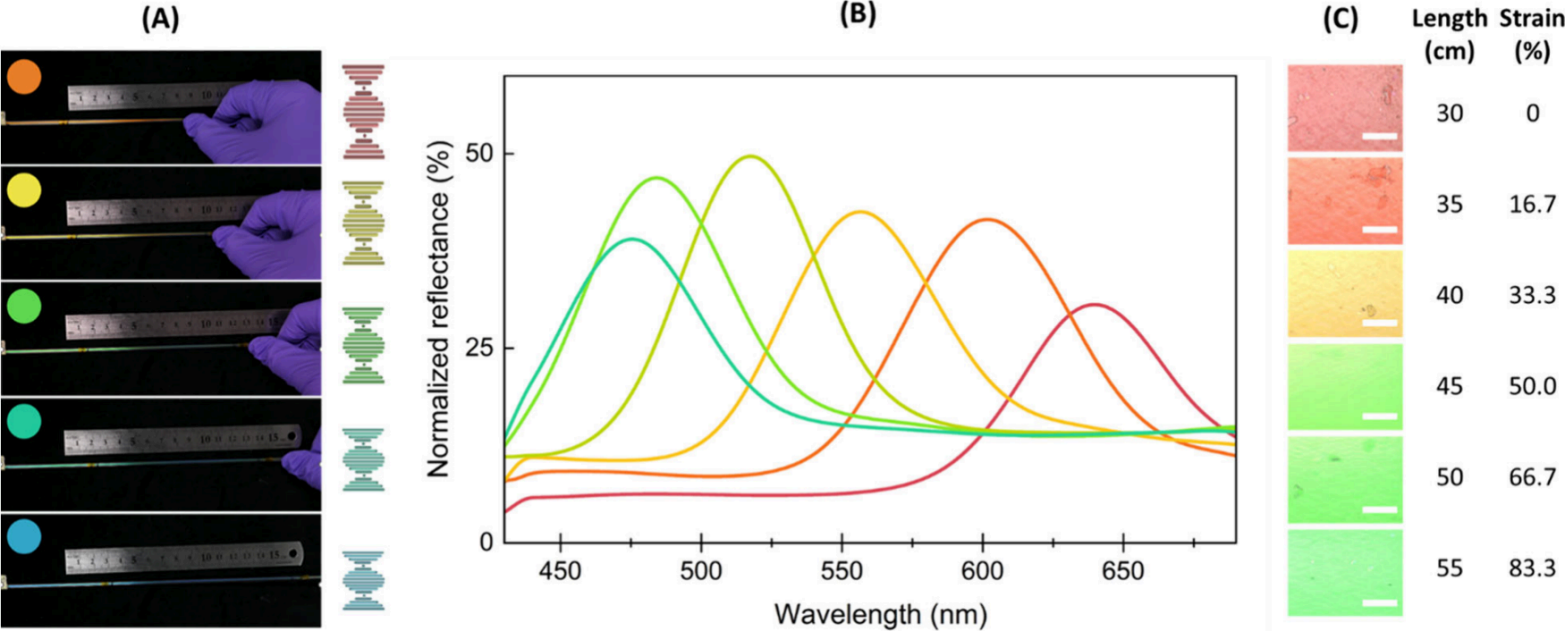
(A) Stretching process of the HPC-tubing system and schematic representation of the pitch of the cholesteric HPC changing upon stretching. (B) Normalized reflection spectra and (C) optical microscopy images of the HPC liquid crystals inside the elastomeric tube (scale bar 100 μm) at strain % deformation points.

When dissolved in water, HPC can spontaneously organize into the cholesteric liquid crystal phase. After being filled with 64.5 wt % HPC solutions, the flexible and transparent Tygon tubes created a binary composite demonstrating the reflective color of HPC. We investigated the colorimetric response of these flexible HPC filaments by applying stretching deformation, Figure 1(A). Starting from a red filament, as the strain was increased, a visible blue shift was observed.

Assuming the incident light is perpendicular to the cholesteric vector of the HPC mesophase, the wavelength of the reflected color can be estimated using de Vries’s eq (eq 1). The cholesteric pitch *p* and the average refractive index *n*_*av*_ both contribute to the macroscopic coloration of the tube. *n*_*av*_ can be calculated with eq 2.
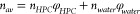
2where φ represents the content of HPC, the average refractive index of HPC is *n*_*HPC*_ = 1.475, and refractive index of water is *n*_*water*_ = 1.333. Thus, the overall refractive index of the system *n*_*a*__v_ = 1.425.

**Figure 2:**
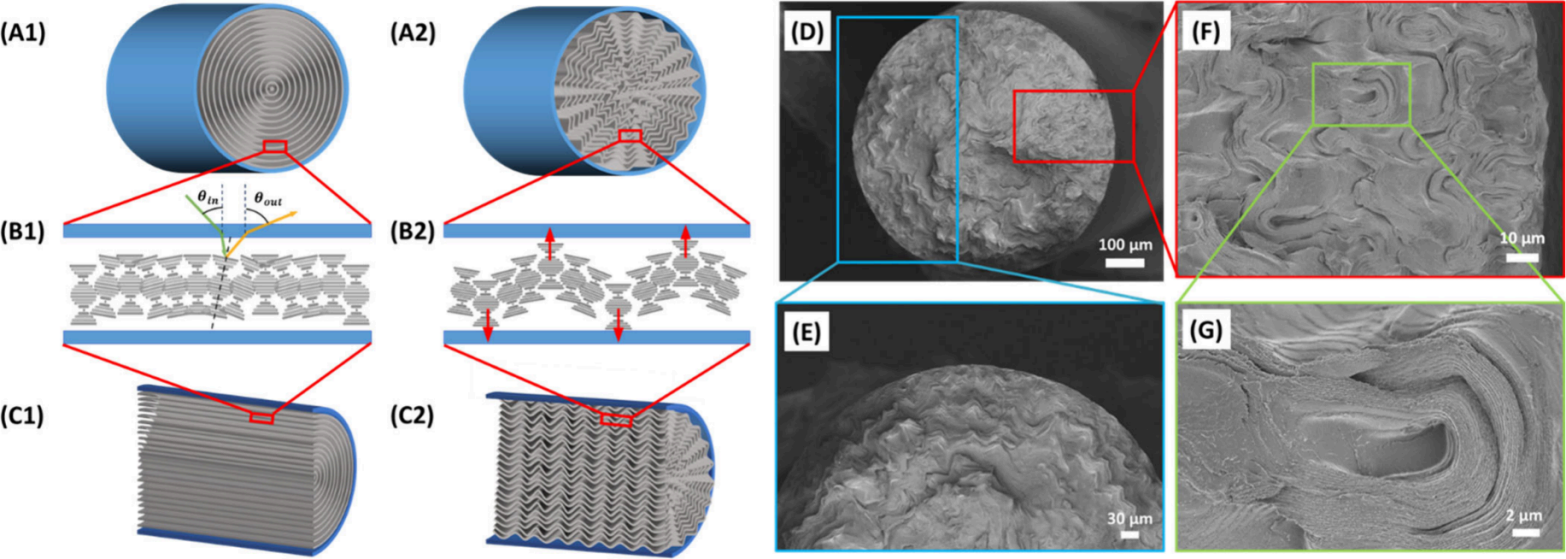
(A–C) Schematics of the idealized cholesteric order of the HPC inside the elastomeric tubing with quasilayers and their radial distribution numbers 1) and 2) indicate before and after dehydration of the cholesteric phase, respectively, following cross-section of both diameter and length. (D–G) SEM images of the cross-section of dried HPC in the tubing.

Equation 1 is valid for a liquid crystal in a hypothetical infinite cholesteric order with uniaxial orientation of the cholesteric quasi-layers. In real cases, however, the orientation of the cholesteric liquid crystals is not uniform throughout the HPC solution as the millimeter-scale volume allows mobility. It has been reported that when HPC is coupled between flexible sheets, there are numerous microdomains with distributed tilting angles emerge.^24^ These micro domains lead to angular reflections, contributing to the wavelength selective scattering, as described in Ferguson’s equation:^26,37,38^
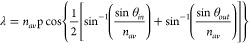
3where *θ*_*in*_ and *θ*_*out*_ represent the angle of incidence and angle of reflection, respectively, as depicted in Figure 2(B1).

In our system, the cholesteric domains within the tubular confinement organize themselves along the central axis of the tube with a radial distribution, Figure 2(A1). From a macroscopic perspective, each infinitesimally small area along the circumference of the tube can be treated as a planar structure, and thus, it also follows eq 3, as shown in Figure 2(B1).

In the stretchable filament configuration, the HPC inside the tubing was kept in the viscous liquid phase which created a liquid crystalline system deformable upon application of mechanical force. While the viscous behavior and the stickiness of the bare HPC solutions are not suitable for the direct application of stretch or compression forces, when it is encapsulated with the Tygon tubing, the liquid crystalline phase follows the mechanical behavior of the encasing material creating mechanochromic sensors. In such a configuration, the cholesteric pitch becomes dependent on the applied strain force, resulting in significant color change.^26,31^

It is also worth mentioning that the HPC/Tygon tube construction presented in this work has dual mechanochromic response against both compression and stretching deformations. When compression forces are applied, it can cause a spontaneous blueshift in the cholesteric pitch which can be detected visually in agreement with the previous studies where HPC was used as an optical compression sensor, Figure S2, Video 2. Interestingly, when the encapsulated HPC filaments were stretched along the longitudinal axis of the filament, a distinct blueshift was observed, as shown in Figure 1(A). During the stretching process, the wall of the Tygon tubing compresses the HPC solution, deforming the cholesteric order in the longitudinal direction causing a blueshift of the reflected coloration, Figure 1(B).

Such an elongation-dependent color shift can be measured spectroscopically, by monitoring the reflection signal from the central area within the 10 cm line of the HPC tubing system, as shown in Figure 1(C) and (D). Stretching the HPC tube from 30 to 55 cm, the microscopic images represent a uniform color change from red (640 nm) to green (475 nm). It is worth noting that, by visual observation and spectral analysis, the reflective coloration of the HPC tubes was visible brighter after stretching. Such an increase in the reflection response can be attributed to an enhancement in the cholesteric order alignment with mechanical stretching. The presence of this enhanced alignment due to stretching was also confirmed via observation of increased reflection intensity, seen in peaks from 640 to 517 nm, Figure 1(C). As the diameter of the tubes decreased with mechanical force, the volume of the HPC solution in the observed region was also reduced. Therefore, with less reflective light being captured, a decrease in the peak intensity at 483 and 475 nm was expected.

Application of axial tensile (Figure 1) and compressive stresses (Figure S1) on the tube wall both caused a blueshift in the visible range, indicating the arrangement of HPC liquid crystals in the tube allows movement in both directions along the longitudinal axis perpendicular to the longitudinal axis without interrupting the cholesteric structure. In the filament configuration, the quasi-layered structure of the HPC aligns radially following the tube geometry as shown in Figure 2(A1), (B1), and (C1). To confirm this, the HPC solutions inside the Tygon tubes were dried at 70 °C and cross-sectioned for further microstructural imaging. The SEM images provided in Figure 2(D) and (E), reveal the wrinkled concentric radial distribution. Upon drying, the radially distributed wrinkles were emerged due to the confinement effects as a result of strain imposed instabilities in CLC systems, which is known as the Helfrich-Hurault (HH) elastic instability^20,24,36,39^ To maintain the preferred quasi-layered spacing, HH effect occurs by relaxing the structure while creating undulations in the CLC structure in response to geometric incompatibilities.^36^ It is well established that the HH effect can be induced by electromagnetic field distortion,^40−44^ and recent studies have shown that this effect can also arise from strain instabilities caused by dehydration.^20,24,39^ We also identified irregular curvatures toward the center of the filaments and more defined wrinkles toward the edge of the filaments in the cross-section as shown in Figure 2 F) and (G). Such change in the wrinkling density emerges from the stresses caused along the transverse and longitudinal directions, as shown in Figure 2(A2), (B2), and (C2).^24^

**Figure 3:**
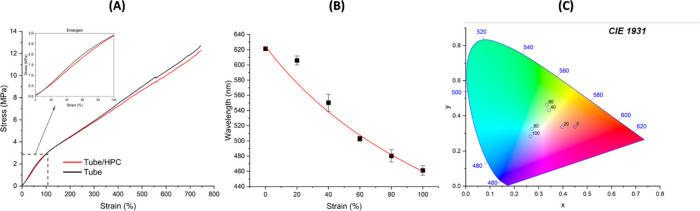
(A) Tensile stress and strain behavior of empty tubing and HPC-encapsulated tubing (tube/HPC). (B) The correlation between wavelength of reflected light of tubing/HPC and applied strain, where the black squares are experimental data points and the red line is a power-law fitted curve. (C) CEI 1931 plot of measured reflectance from tubing/HPC at different strain values. The D65 standard was used to predict the color in daylight.

Tensile tests were performed to determine the relationship between the stress–strain and the colorimetric changes of the flexible HPC tubing system. Figure 3(A) indicates the stress–strain behavior of the empty tubing and encapsulated HPC tubes. Comparing the tensile deformation behavior between the hollow elastomer tube and the elastomer tube filled with HPC mesophase, it can be concluded that the presence of the HPC solution does not significantly alter the mechanical deformation characteristics of the tubes. Both samples demonstrated high elongations up to 800% strain and a linear elastic region up to around 100% strain. This suggests that the addition of the HPC mesophase maintains the mechanical integrity of the elastomer tubing during tensile loading. In the linear elastic region, as the tubes were stretched, the tube walls were in a triaxial stress state, including tensile stress along the tube axial (σ_A_), radial stress (σ_R_) and circumferential stress (σ_C_), as shown in Figure S2.^45−49^ The longitudinal stretch applied induces the circumferential tensile stress and radial compressive stress (Figures S3 and S4). As discussed previously, the radial compressive stress acting on the tube walls increases the confinement of the HPC and compresses it longitudinally, resulting in a reduced helicoidal pitch. Both the manual stretching and tensile testing of the HPC in the elastomeric tube, caused a blueshift in the color response as expected.

To understand how coloration is varied by applied strain, we correlated the reflective wavelength as a function of strain, Figure 3(B). Based on the assumption that the volume of the Tube/HPC system remains the same during stretching, the relationship between the reflected wavelength (λ) and the strain (ε) follows a power law function:
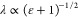
4

For the detailed calculation process, please refer to the Section S5. As indicated by the red fitting curve in Figure 3(B), the experimental data closely matched the theoretical model described by eq 4 with a coefficient of determination (R^2^) greater than 96%, confirming a strong correlation between the reflected wavelength and the applied strain, Figure S5.

Additionally, the variation in chromaticity (D65) with strain can be tracked using the CIE 1931 plot, as shown in Figure 3(C). The cyclic loading experiments indicate the excellent consistency and negligible relaxation of the HPC/Tube system, further supported by the data presented in Figure S6(A) and (B). On the other hand, the system also shows a reduction of angular dependency due to its concentric circular confinement, Figure S7. Both demonstrate that the color shift in the system is highly predictable based on the mechanical behavior of the encapsulating tube.

## Conclusion

In conclusion, we fabricated a stretchable filament by encapsulating the cholesteric mesophases of the HPC in thermoplastic elastomer Tygon tubing and demonstrated its mechanochromic sensing ability. Under axial instantaneous tensile loading and release, the HPC/tubing system exhibited a significant blueshift of the reflected color following a power-law relationship, without substantially altering the mechanical properties of the tubing. Upon release, the HPC/Tube system recovered perfectly returning to its original color following the relaxation of the tube. Understanding the boundary conditions between the elastomer tube and cholesteric phase and their relaxation behavior poses an intriguing question to follow this work. As observed in the dehydrated HPC filament peeled from the tube, we found radial confinement of dehydrated quasi-layers along the central axis of the tubing, which exhibited continuous wrinkles resulting from the HH effect. Such unique alignment allows the CLC mesophase in the system to respond uniformly and flexibly to both compressive and tensile stresses.

Beyond expanding our understanding of mechanically induced changes in the cholesteric mesophase and its dynamic coloration, this prototype composite system indicates potential for real-world use. It is worth noting that simply filling HPC into commercially available elastic tubing only serves as a proof-of-concept for this system. However, for real-world applications, more scalable and efficient methods would be preferred. Instead of filling bulk tubes with high-pressures, approaches such as cross-linking HPC into elastomers or using coaxial fiber extrusion can be considered, which would simultaneously produce an encapsulated filament. Those approaches would streamline manufacturing and enhance the practical viability of the system for broader applications such as functional smart wearables and color-shifting materials for arts.

## Supporting Information Available

Section S1. Refractive index of HPC (Figure S1). Section S2: Mechanochromic response under compression (Figure S2). Section S3: Force analysis of Tubing (Figures S3–S4). Section S4: Comparation of tensile test (Table S1). Section S5: Relationship between reflected wavelength of tubing/HPC and strain (Figure S5). Section S6: Cyclic loading and time-dependent relaxation (Figure S6). Section S7: Angular dependence (Figure S7). (PDF)
Video 1: Stretching Tube/HPC system (MP4)
Video 2: Compressing Tube/HPC system (MP4)

## References

[ref1] Boott C. E.; Tran A.; Hamad W. Y.; Maclachlan M. J. Cellulose Nanocrystal Elastomers with Reversible Visible Color. Angew. Chem. 2020, 132 (1), 232–237. 10.1002/ange.201911468. 31663249

[ref2] Kose O.; Tran A.; Lewis L.; Hamad W. Y.; MacLachlan M. J. Unwinding a spiral of cellulose nanocrystals for stimuli-responsive stretchable optics. Nat. Commun. 2019, 10 (1), 510 10.1038/s41467-019-08351-6. 30705267 PMC6355765

[ref3] Liu Y.; Wu P. Bioinspired Hierarchical Liquid-Metacrystal Fibers for Chiral Optics and Advanced Textiles. Adv. Funct. Mater. 2020, 30 (27), 2002193 10.1002/adfm.202002193.

[ref4] Kizhakidathazhath R.; Geng Y.; Jampani V. S. R.; Charni C.; Sharma A.; Lagerwall J. P. F. Facile Anisotropic Deswelling Method for Realizing Large-Area Cholesteric Liquid Crystal Elastomers with Uniform Structural Color and Broad-Range Mechanochromic Response. Adv. Funct. Mater. 2020, 30 (7), 1909537 10.1002/adfm.201909537.

[ref5] Geng Y.; Lagerwall J. P. F. Multiresponsive Cylindrically Symmetric Cholesteric Liquid Crystal Elastomer Fibers Templated by Tubular Confinement. Advanced Science 2023, 10 (19), 2301414 10.1002/advs.202301414. 37186075 PMC10323659

[ref6] Hisano K.; Kimura S.; Ku K.; Shigeyama T.; Akamatsu N.; Shishido A.; Tsutsumi O. Mechano-Optical Sensors Fabricated with Multilayered Liquid Crystal Elastomers Exhibiting Tunable Deformation Recovery. Adv. Funct. Mater. 2021, 31 (40), 2104702 10.1002/adfm.202104702.

[ref7] Nam S.; Jung W.; Shin J. H.; Choi S. S. Omnidirectional color wavelength tuning of stretchable chiral liquid crystal elastomers. Light: Science & Applications 2024, 13 (1), 114 10.1038/s41377-024-01470-w. PMC11109264 38773092

[ref8] Nishio Y.; Susuki S.; Takahashi T. Structural Investigations of Liquid-Crystalline Ethylcellulose. Polym. J. 1985, 17 (6), 753–760. 10.1295/polymj.17.753.

[ref9] De Vries H. Rotatory power and other optical properties of certain liquid crystals. Acta Crystallogr. 1951, 4 (3), 219–226. 10.1107/S0365110X51000751.

[ref10] Kuo C.; Lin Y.; Huang T.; Liu C. Thermochromic Liquid-Crystalline Elastomers Featuring a Predesigned Hybrid Architecture. ACS Applied Polymer Materials 2024, 6 (15), 9080–9087. 10.1021/acsapm.4c01347.

[ref11] Almeida P. L.; Tavares S.; Martins A. F.; Godinho M. H.; Cidade M. T.; Figueirinhas J. L. Cross-linked hydroxypropylcellulose films: mechanical behaviour and electro-optical properties of PDLC type cells. Opt. Mater. 2002, 20 (2), 97–100. 10.1016/S0925-3467(02)00051-4.

[ref12] Brannum M. T.; Steele A. M.; Venetos M. C.; Korley L. T. J.; Wnek G. E.; White T. J. Light Control with Liquid Crystalline Elastomers. Advanced Optical Materials 2019, 7 (6), 1801683 10.1002/adom.201801683.

[ref13] Coates D. Development and applications of cholesteric liquid crystals. Liq. Cryst. 2015, 42 (5–6), 653–665. 10.1080/02678292.2015.1020454.

[ref14] Radka B. P.; Lee K. M.; Godman N. P.; White T. J. Electro-optic characteristics of stabilized cholesteric liquid crystals with non-liquid crystalline polymer networks. Soft Matter 2022, 18 (15), 3013–3018. 10.1039/D2SM00203E. 35355040

[ref15] Bampidis V.; Azimonti G.; Bastos M. d. L.; Christensen H.; Dusemund B.; Kos Durjava M.; Kouba M.; López-Alonso M.; López Puente S.; Marcon F.; Mayo B.; Pechová A.; Petkova M.; Ramos F.; Sanz Y.; Villa R. E.; Woutersen R.; Bories G.; Gropp J.; Nebbia C.; Innocenti M. L.; Aquilina G. Safety and efficacy of hydroxypropyl cellulose for all animal species. EFSA Journal 2020, 18 (7), e06213 10.2903/j.efsa.2020.6213. 32760469 PMC7393479

[ref16] Fukui E.; Uemura K.; Kobayashi M. Studies on applicability of press-coated tablets using hydroxypropylcellulose (HPC) in the outer shell for timed-release preparations. Journal of controlled release 2000, 68 (2), 215–223. 10.1016/S0168-3659(00)00261-3. 10925130

[ref17] Joshi G.; Rana V.; Naithani S.; Varshney V. K.; Sharma A.; Rawat J. S. Chemical modification of waste paper: An optimization towards hydroxypropyl cellulose synthesis. Carbohydr. Polym. 2019, 223, 115082 10.1016/j.carbpol.2019.115082. 31426970

[ref18] Burdock G. A. Safety assessment of hydroxypropyl methylcellulose as a food ingredient. Food Chem. Toxicol. 2007, 45 (12), 2341–2351. 10.1016/j.fct.2007.07.011. 17723258

[ref19] Godinho M. H.; Gray D. G.; Pieranski P. Revisiting (hydroxypropyl) cellulose (HPC)/water liquid crystalline system. Liq. Cryst. 2017, 1–13. 10.1080/02678292.2017.1325018.

[ref20] Ren H.; Balcerowski T.; Dumanli A. G. Achieving a full color palette with thickness, temperature, and humidity in cholesteric hydroxypropyl cellulose. Frontiers in Photonics 2023, 4, na 10.3389/fphot.2023.1134807.

[ref21] Werbowyj R. S.; Gray D. G. Liquid Crystalline Structure In Aqueous Hydroxypropyl Cellulose Solutions. Mol. Cryst. Liq. Cryst. 1976, 34 (4), 97–103. 10.1080/15421407608083894.

[ref22] Chiba R.; Ito M.; Nishio Y. Addition effects of imidazolium salts on mesophase structure and optical properties of concentrated hydroxypropyl cellulose aqueous solutions. Polym. J. 2010, 42 (3), 232–241. 10.1038/pj.2009.338.

[ref23] Nakamura A.; Yamane N.; Murakami K. Development of smart window with hydroxypropyl cellulose-acrylamide hydrogel: Effect of hydroxypropyl cellulose molecular weight on light scattering property. Optik 2023, 294, 171464 10.1016/j.ijleo.2023.171464.

[ref24] Balcerowski T.; Ozbek B.; Akbulut O.; Dumanli A. G. Hierarchical Organization of Structurally Colored Cholesteric Phases of Cellulose via 3D Printing. Small 2023, 19 (8), 2205506 10.1002/smll.202370049. 36504424

[ref25] Yamagishi T. A.; Guittard F.; Godinho M. H.; Martins A. F.; Cambon A.; Sixou P. Comparison of thermal and cholesteric mesophase properties among the three kind of hydroxypropylcellulose (HPC) derivatives. Polym. Bull. 1994, 32 (1), 47–54. 10.1007/BF00297413.

[ref26] Kamita G.; Frka-Petesic B.; Allard A.; Dargaud M.; King K.; Dumanli A. G.; Vignolini S. Biocompatible and Sustainable Optical Strain Sensors for Large-Area Applications. Advanced Optical Materials 2016, 4 (12), 1950–1954. 10.1002/adom.201600451.

[ref27] Kamita G.; Vignolini S.; Dumanli A. G. Edible cellulose-based colorimetric timer. Nanoscale Horizons 2023, 8, 887 10.1039/D3NH00006K. 37066860 PMC10291961

[ref28] Yi H.; Lee S.-H.; Kim D.; Jeong H. E.; Jeong C. Colorimetric Sensor Based on Hydroxypropyl Cellulose for Wide Temperature Sensing Range *Sensors* [Online]. Sensors 2022, 22, 886 10.3390/s22030886. 35161632 PMC8839604

[ref29] Leal-Junior A.; Rocha H.; Almeida P. L.; Marques C. Force Estimation With Sustainable Hydroxypropyl Cellulose Sensor Using Convolutional Neural Network. IEEE Sensors Journal 2024, 24 (2), 1366–1373. 10.1109/JSEN.2023.3332659.

[ref30] Wei J.; Aeby X.; Nyström G. Printed Structurally Colored Cellulose Sensors and Displays. Advanced Materials Technologies 2023, 8 (1), 2200897 10.1002/admt.202200897.

[ref31] Barty-King C. H.; Chan C. L. C.; Parker R. M.; Bay M. M.; Vadrucci R.; De Volder M.; Vignolini S. Mechanochromic, Structurally Colored, and Edible Hydrogels Prepared from Hydroxypropyl Cellulose and Gelatin. Adv. Mater. 2021, 33 (37), 2102112 10.1002/adma.202102112. 34323315 PMC11468689

[ref32] Shi H.; Deng Y.; Shi Y. Cellulose-Based Stimuli-Responsive Anisotropic Hydrogel for Sensor Applications. ACS Applied Nano Materials 2023, 6 (13), 11524–11530. 10.1021/acsanm.3c01551.

[ref33] Liang H.; Bay M. M.; Vadrucci R.; Barty-King C. H.; Peng J.; Baumberg J. J.; De Volder M. F. L.; Vignolini S. Roll-to-roll fabrication of touch-responsive cellulose photonic laminates. Nat. Commun. 2018, 9 (1), 4632 10.1038/s41467-018-07048-6. 30401803 PMC6219516

[ref34] Prince E.; Wang Y.; Smalyukh I. I.; Kumacheva E. Cylindrical Confinement of Nanocolloidal Cholesteric Liquid Crystal. J. Phys. Chem. B 2021, 125 (29), 8243–8250. 10.1021/acs.jpcb.1c04387. 34259528

[ref35] Ma L.; Li S.; Li W.; Ji W.; Luo B.; Zheng Z.; Cai Z.; Chigrinov V.; Lu Y.; Hu W.; Chen L. Rationally Designed Dynamic Superstructures Enabled by Photoaligning Cholesteric Liquid Crystals. Advanced Optical Materials 2015, 3 (12), 1691–1696. 10.1002/adom.201500403.

[ref36] Blanc C.; Durey G.; Kamien R. D.; Lopez-Leon T.; Lavrentovich M. O.; Tran L. Helfrich-Hurault elastic instabilities driven by geometrical frustration. Rev. Mod. Phys. 2023, 95 (1), 015004 10.1103/RevModPhys.95.015004.

[ref37] Werbowyj R. S.; Gray D. G. Optical properties of hydroxypropyl cellulose liquid crystals. I. Cholesteric pitch and polymer concentration. Macromolecules 1984, 17 (8), 1512–1520. 10.1021/ma00138a016.

[ref38] Fergason J. L. Cholesteric Structure-1 Optical Properties. Molecular Crystals 1966, 1 (2), 293–307. 10.1080/15421406608083274.

[ref39] Chan C. L. C.; Bay M. M.; Jacucci G.; Vadrucci R.; Williams C. A.; van de Kerkhof G. T.; Parker R. M.; Vynck K.; Frka-Petesic B.; Vignolini S. Visual Appearance of Chiral Nematic Cellulose-Based Photonic Films: Angular and Polarization Independent Color Response with a Twist. Adv. Mater. 2019, 31 (52), 1905151 10.1002/adma.201905151. 31736173

[ref40] Helfrich W. Deformation of Cholesteric Liquid Crystals with Low Threshold Voltage. Appl. Phys. Lett. 1970, 17 (12), 531–532. 10.1063/1.1653297.

[ref41] Hurault J. P. Static distortions of a cholesteric planar structure induced by magnetic or ac electric fields. J. Chem. Phys. 1973, 59 (4), 2068–2075. 10.1063/1.1680293.

[ref42] Frka-Petesic B.; Guidetti G.; Kamita G.; Vignolini S. Controlling the Photonic Properties of Cholesteric Cellulose Nanocrystal Films with Magnets. Adv. Mater. 2017, 29 (32), 1701469 10.1002/adma.201701469. 28635143

[ref43] Lavrentovich O. D.; Yang D. K. Cholesteric cellular patterns with electric-field-controlled line tension. Phys. Rev. E 1998, 57 (6), R6269–R6272. 10.1103/PhysRevE.57.R6269.

[ref44] Ishikawa T.; Lavrentovich O. D. Undulations in a confined lamellar system with surface anchoring. Phys. Rev. E 2001, 63 (3), 030501 10.1103/PhysRevE.63.030501. 11308620

[ref45] Guo W.; Gao Y.; Wu X.; Liu S.; Fan P. Elastoplastic analysis of the enhancing mechanism of tensile performance of ductile thin-walled circular tubes with internal support. Thin-Walled Structures 2022, 171, 108694 10.1016/j.tws.2021.108694.

[ref46] Vincenzo V. Circular Cylinders and Pressure Vessels. Springer International Publishing: Cham, Switzerland, 2014.

[ref47] Gao Y.; Fei S.; Qian X.; Pengxian F.; Lei G.; Linyue B.; Xintong Y.; Huishuang Z. Mechanical analysis of enhancement in tensile performance of thin-walled circular tubes by internal support. Advances in Mechanical Engineering 2021, 13 (7), 168781402110327 10.1177/16878140211032794.

[ref48] Zhou M.; Xu L.; Tao M.; Fan J.; Hajjar J. F.; Nie J. Experimental study on confining-strengthening, confining-stiffening, and fractal cracking of circular concrete filled steel tubes under axial tension. Engineering Structures 2017, 133, 186–199. 10.1016/j.engstruct.2016.12.008.

[ref49] Bellarby J. Chapter 9. Tubing Stress Analysis. In Developments in Petroleum Science; Bellarby J., Ed.; Elsevier, 2009; Vol. 56, pp 473–556.

