# Supplementary material for: Stretchable Cellulosic Cholesteric Liquid Crystal Filaments with Color Response
Source: ACS Appl Polym Mater. 2025 Mar 26;7(7):4093–8. doi: 10.1021/acsapm.4c02719 (PMC11997954; doi:10.1021/acsapm.4c02719)
Supplement: Supplementary file 1 — ap4c02719_si_001.pdf [file ap4c02719_si_001.pdf]

Supporting Information

Stretchable cellulosic cholesteric liquid crystal filaments with colour response

Hongning Ren, [a,b] Ifeoluwa Omolola Sodipo [a,b] and Ahu Gümrah Dumanli *[a,b]

[a] *Department of Material, the University of Manchester, M13 9PL.*
[b] *Henry Royce Institute, the University of Manchester, M13 9PL.*
* *ahugumrah.parry@manchester.ac.uk*

Section S1. Refractive index of HPC

The average refractive index (RI) of HPC has been carefully measured using A. KRÜSS Optronic Analogue AR3 Abbe Refractometer. Before measurement, A series of HPC solution of different mass ratio were prepared with MilliQ water 8.2 MΩ cm, Suez Fusion 320. To extract the RI, the data points were linear fitted using OriginPro 2021b.

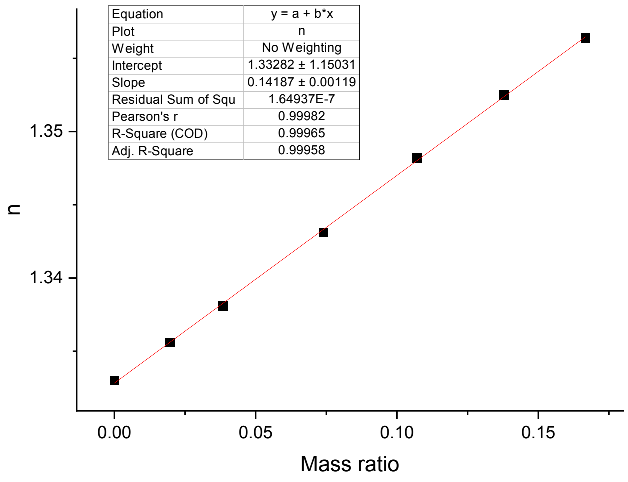

Figure S1. Linear fitting of the RI measurement of HPC solutions.

By linear fitting, we can extract the RI from both intercept and slope,

$$n_{HPC}=1.475,$$

Where n_{HPC} is the average of the ordinary and extraordinary refractive indices of HPC.

The average refractive index of HPC solution can be calculated,

$$n_{av} = n_{HPC}\varphi_{HPC} + n_{water}\varphi_{water}$$

$n_{water} = 1.333$

$$\varphi_{HPC} = 0.645$$

$$\varphi_{water} = 0.355$$

Here we can get

$$n_{av} = 1.42459 \approx 1.425$$

Section S2 Mechanochromic response under compression

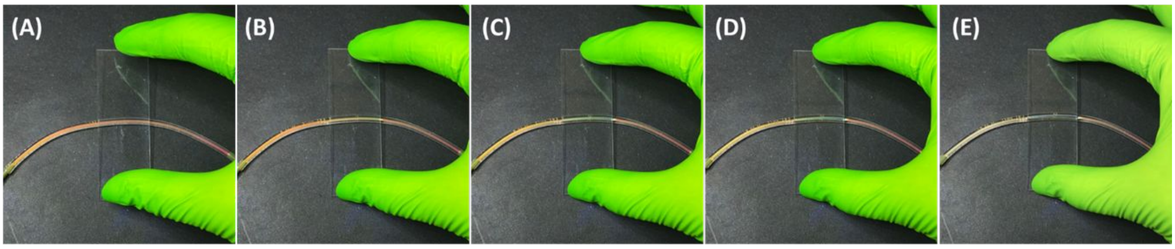

Mass (g)	0	300	683	1116	1883
Force (N)	0	2.94	6.70	10.94	18.47

Figure S2 (A) The cholesteric HPC is confined in the elastomer tubing system without compression. (B-E) The blue shift colour response of the HPC-tubing system when compressed following increased loading force. The HPC-tubing system was placed between two glass slides. The force was measured by applying the load on the glass slides while manually compressing on the balance and visible colour shift was recorded using an iPhone 11 camera. The figure is corresponding to Video S2.

Section S3 Force analysis of Tubing

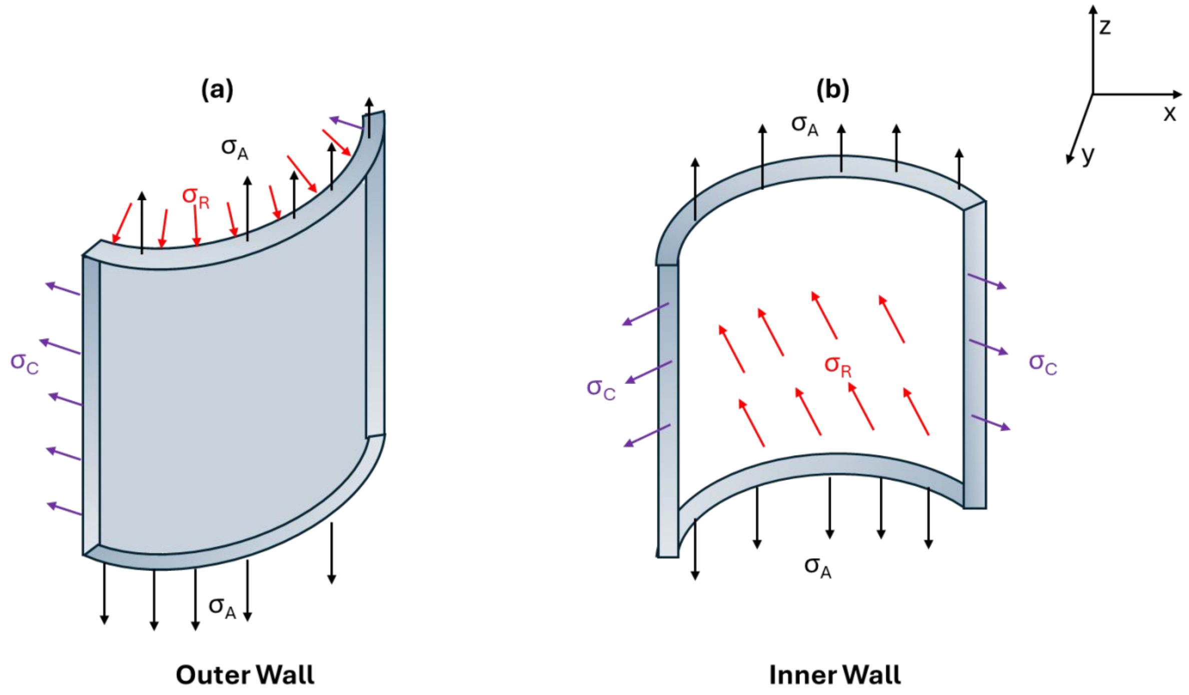

Figure S3 Triaxial stress state of the tube viewed from the (a) outer wall and the (b) inner wall. The axial stress circumferential stress and radial stress all act in all principal directions.

Below gives a clarification for statements made on triaxial stress states:

The inner wall is in triaxial stress states as all principal directions have non-zero values, as shown in the Figure below. Axial stress (σ_A, tensile) acts in the z-direction. Internal pressure (p) caused by the HPC filler acts on the inner surface (σ_R = -p) and

acts perpendicular to the tube's axis. This pressure induces the circumferential stress (σ_C), also known as the hoop or the tangential stress, acting tangentially to the tube z-axis.

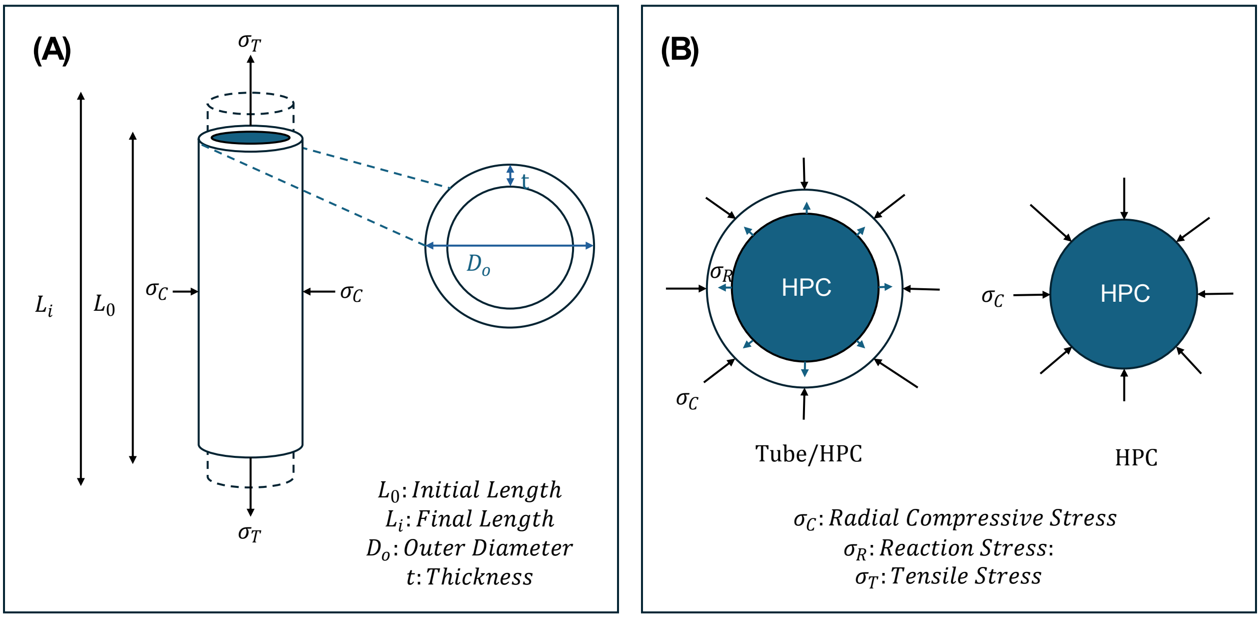

Figure S4 (A) Tube geometry and stress distribution as axial tensile stress (σ_T) is applied on the tube. (B) Stress distribution in the radial cross-section of the HPC encapsulated tube as a tensile stress is applied. A radial compressive stress (σ_C) is induced, compressing the HPC and creating a pressure reaction stress (σ_R).

During the tensile test, as the axial tension increases and the tube is stretched, there is a lateral contraction of the tube due to the Poisson effect. This leads to increasing confinement of the HPC, causing structural changes and altering the colour.

Section S4 Comparation of tensile test

Table S1 Calculated mechanical properties of empty tubing (tube) and HPC-encapsulated tubing (tube/HPC).

Sample	Average Strain at break (%)	Average Stress at break (MPa)	Average Young's Modulus (MPa)
Tube	679 ± 106	11.8 ± 1.4	3.24 ± 0.10
Tube/ HPC	722 ± 27	11.8 ± 0.5	3.05 ± 0.01

Section S5 Relationship between reflected wavelength of tubing/HPC and strain

As we assume the volume (V) of the whole fibre does not change during stretching, only the cross-section area (S) and length (L) changed, we can get

$$V = L_o S_o = LS$$

Because $S = \pi R^2$, where R represents the radius of the cylinder,

Here we can get the transformation between L, S and R

$$\frac{L_o}{L} = \frac{S}{S_o} = \frac{R^2}{{R_o}^2}$$

And the strain (ε) is the difference between L1 and L2

$$\varepsilon = \frac{L - L_o}{L_o}$$

$$= \frac{L}{L_o} - 1$$

$$= \frac{{R_o}^2}{R^2} - 1$$

$$\frac{R_o}{R} = \sqrt{\varepsilon + 1}$$

$$R = \frac{R_o}{\sqrt{\varepsilon + 1}}$$

As the reflective wavelength (λ)can be calculated from the cholesteric pitch (P), according to the de Vries's equation,

$$\lambda = n_{av} \cdot p \cdot \cos\theta$$

Where n is the average refractive index and θ is the observation angle.Since we assume the number of pseudo layers does not change during the spontaneous change of stretching, p is changing following R. Thus, we can get

$$p \propto R$$

$$\lambda \propto R = \frac{R_o}{\sqrt{\varepsilon + 1}}$$

After simplifying, we can get the

$$\lambda \propto (\varepsilon + 1)^{-\frac{1}{2}}$$

According to the relationship, we did a two-parameter power-low fitting which achieved the coefficient of determination (R^2) over 97%, where the power number = -0.44 is close to our calculation of -0.5. By fixed powder low fitting using -0.5, we also get an acceptable R^2 greater than 96%. Therefore, we can confirm a strong

correlation between the reflected wavelength and the applied strain following $\lambda \propto (\varepsilon + 1)^{-1/2}$

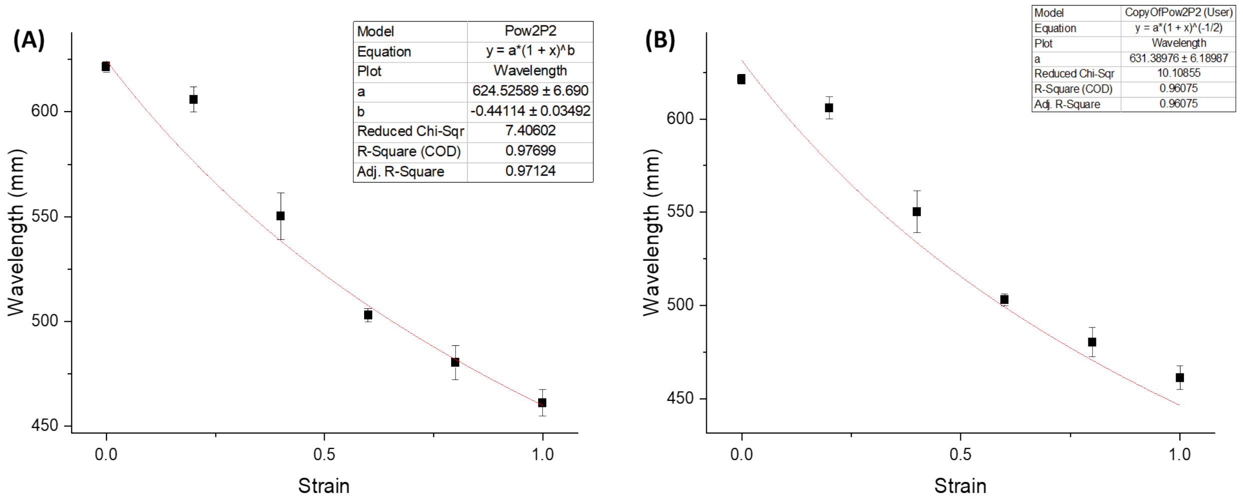

Figure S5 Curve fitting of the wavelength of reflected light of tubing/HPC and strain by (A) two-parameter power-low fitting and (B) single-parameter power-low fitting with fixed power of -0.5.

Section S6 Cyclic loading and relaxation of the cholesteric phase

As shown in Fig. S6 (A), the cyclic loading experiment was performed using the sample of HPC/Tube system by carefully stretching the filaments up to 100% strain in 10 seconds, relaxing the filament for 1 minute and repeating the strain test five times. To prepare the sample, HPC solutions (64.5wt. %) were extruded into a 40 cm long tube. And to exclude the influence of both sides, only the central 30 cm was used for observation.

The possible relaxation of the stretched HPC/Tube system was analysed using a 45 cm sample containing a 64 wt.% solution. The original reflectance of this sample was recorded as 675 nm (reflectance maxima). This tube system was then stretched to 55 cm (reflective wavelength of 630 nm) and securely fixed. The time-dependent reflectance wavelength was recorded every 2 minutes within a 10-minute overall experiment time. The time-dependent reflectance measurements are given in Fig. S6 (B) and reveal a slight redshift occurring during the experimentation time, indicating relaxation of the cholesteric phase. Notably, the relaxation process gradually slowed and did not return to the original colouration after 10 minutes, signifying the applied stress also caused an overall shift in the cholesteric periodicity. While the HPC/Tube system is a binary element composite, the cholesteric phase follows the mechanical deformation behaviour of the elastomeric tubing. While there is evidence of a slight shift (1.78%) at the reflectance maxima and a broader pitch distribution measured after stretching, elucidating the exact origin of this shift could be a highly complex task. The shift we observed could be due to cholesteric phase deformation and inhomogeneities between the tube interface, possible changes in the radial

distribution of the cholesteric phase and the interaction between the cholesteric HPC and the tubing at the contact, such as the presence of the microscopic bubbles as well as the relaxation behaviour of the elastic tubing itself. Such change is highly intriguing to explain in a theoretical framework. However, it is out of the scope of this manuscript.

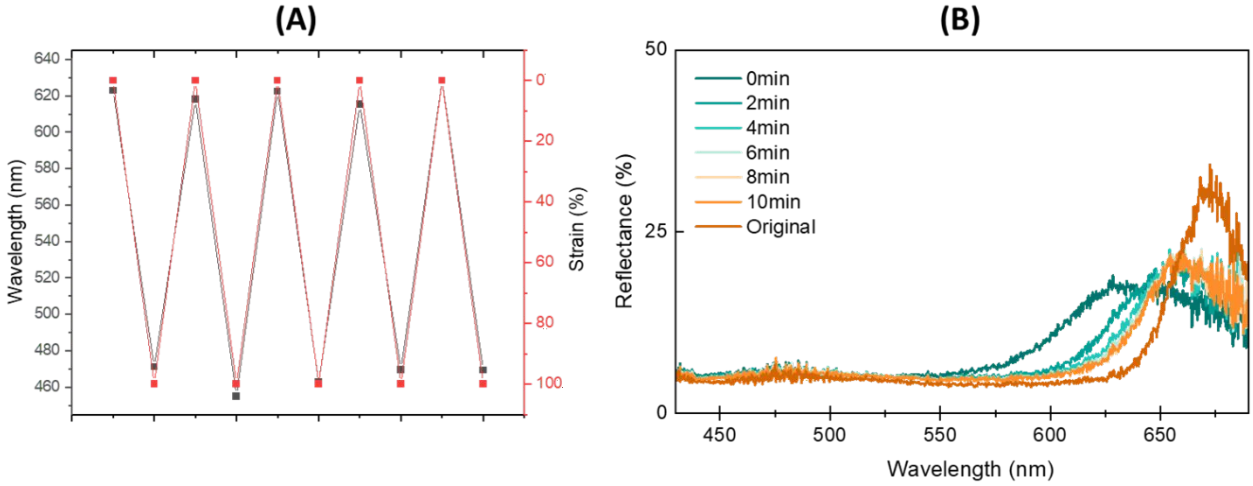

Figure S6 (A)Cyclic tensile loading of HPC/Tube system from the original state to strain of 100%. (B)Relaxation of HPC/Tube system within 10 minutes.

Section S7 Angular dependence

The reflective light can be calculated by using de Vries's equation,

$$\lambda = n_{av} \cdot p \cdot \cos\theta$$

where n is the average refractive index, θ is the angle of reflection with respect to the cholesteric helix axis, and p is the cholesteric pitch.

There are two directions for angular measurement of the tubing system, following the radial direction and the cylinder axis.

In the first case, due to the special concentric confinement as shown in Figure S7 (A), θ is always zero, thus the reflective light will not change by the angle change. In the second case as shown in Figure S7 (B), When we are measuring the same point, following angle change the reflective wavelength get altered. However, the CLC are arranged uniformly following the tubing axis, it has a normal angular dependency. Combining both conditions, and assuming the Helfrich-Hurault instabilities exists due to the confinement, the HPC/tubing system shows an overall reduction of angular of angular dependence.

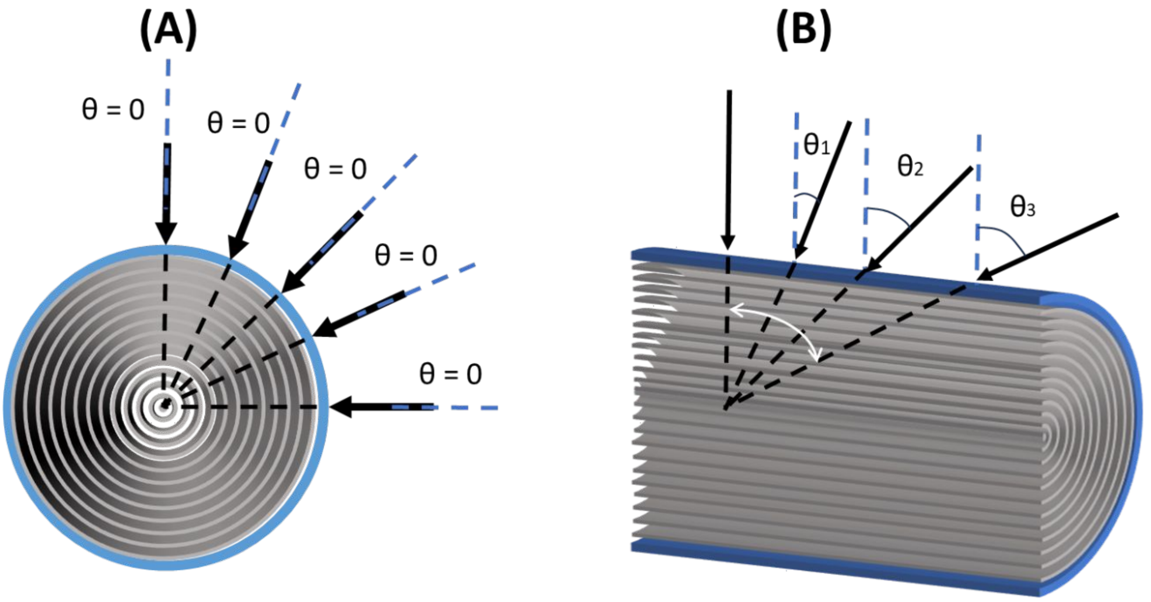

Figure S7 Graphic representation of the cholesteric phase inside the Tygon® tubing. Angular dependence of the visual color (A) in radial direction and (B) along the cylinder axis.
